# Effects of Concurrent Dosing on the Efficacy of Tissue Plasminogen Activator and Deoxyribonuclease in the Treatment of Pleural Infection

**DOI:** 10.7759/cureus.46683

**Published:** 2023-10-08

**Authors:** Vikas Pathak, Lukash Adhikari, Christine Zhou

**Affiliations:** 1 Pulmonary and Critical Care, Virginia Institute of Lung Diseases, Richmond, USA; 2 Internal Medicine, Patan Academy of Health Sciences, Lalitpur, NPL; 3 Pulmonary and Critical Care Medicine, University of Cincinnati Medical Center, Cincinnati, USA

**Keywords:** deoxyribonuclease, pleural infection, dnase, tpa, pleural effusion, empyema

## Abstract

Background: The goal of this study was to evaluate how the administration of concurrent tissue plasminogen activator (tPA) and deoxyribonuclease (DNase) therapy with variable dosing for complicated parapneumonic effusions and empyema affects patient outcomes in an inner-city community hospital.

Methods: This retrospective analysis was performed at an inner-city hospital located in Raleigh, North Carolina. A list of all patients treated with tPA and DNase between July 1, 2015, and December 31, 2017, was generated and screened. Data were collected through a review of past medical records, including demographics, past medical history, and details about their hospital course.

Results: A total of 38 patients were found to have been treated with concurrent tPA and DNase for complicated parapneumonic effusion or empyema. Twenty (52.6%) patients received the full six doses of combined concurrent tPA/DNase. Of the 18 (47.4%) patients who did not receive the full six doses, 11 did not require the full six doses for effusion resolution, and seven had to discontinue therapy due to tube blockage or pain. Only seven (18.4%) patients had complications related to tPA/DNase administration, most commonly pain. Nineteen (50%) patients had complete radiological clearance of effusion, with 13 (34.2%) having partial clearance, and six (15.8%) having no change or worsening of their effusion. Eight (21.1%) patients needed further surgical management of their effusion.

Conclusions: The current most common dosing pattern for combined tPA and DNase therapy of twice daily for three days may not be optimal for all patients. The dosing regimen should be individualized depending on clinical response. Concurrent dosing is safe.

## Introduction

More than 1.5 million people are diagnosed with pleural effusions in the USA annually [1]. It may be related to disorders of the lung or pleura, or a systemic disorder. Most patients typically present with dyspnea, dry cough, and pleuritic chest pain. Pleural effusions are diagnosed best by imaging. In order of feasibility, you can see pleural effusions on bedside ultrasound, chest X-ray, and CT scans. To treat a pleural effusion, it is important to determine the etiology.

In this study, we will focus on primarily parapneumonic exudative effusions. An uncomplicated or simple parapneumonic effusion refers to a free-flowing effusion that is sterile. A complicated parapneumonic effusion (CPPE) or empyema refers to an effusion that forms adjacent to pneumonia that is now infected with bacteria or other microorganisms. An empyema refers to a collection of pus within the pleural space. Effusions can further be classified as loculated vs. un-loculated. Management of CPPE involves prompt antibiotic initiation and drainage of infected pleural fluid. Empyema has a high mortality rate (15%) when compared to a simple pleural effusion, it is therefore very important to initiate early drainage of the pleural fluid and start antibiotics. About one-third of patients fail antibiotic therapy and drainage, requiring surgical drainage, with an average hospital stay of 12 to 15 days, some even as much as a month [2]. In some cases, drainage is made more difficult due to loculations, septations, and increased viscosity of the pleural fluid.

Difficult loculations, septations, and increased viscosity are alleviated with the use of intrapleural tissue plasminogen activator (tPA) and recombinant deoxyribonuclease (DNase). This combination is used in those who fail antibiotic therapy and initial drainage, with hopes to avoid video-assisted thoracic surgery (VATS) and shorten the duration of hospitalization. The use of tPA/DNase in combination has a better effect than either agent alone [2-4]. A large randomized trial [2] was done, which showed a reduction in the need for surgery when tPA was combined with DNase. The current practice is that tPA is dosed at 10 mg and DNase at 5 mg, which are administered via the chest tube or catheter twice daily for three days equaling six doses, given sequentially. This study evaluates if six doses are required for optimal results and if concurrent dosing is safe (primary objective) and efficacious (secondary objective).

This article was previously presented as a meeting abstract at the CHEST Annual Scientific Meeting in San Antonio, Texas on October 10, 2018 [5].

## Materials and methods

Study design

The study was designed as a retrospective study in a cohort of patients who had a pleural infection and received tPA and DNase. It was approved by the WakeMed Institutional Review Board (approval number: 1168410-2). The study period was 2.5 years.

Data collection

All data were collected from electronic medical records. Comprehensive data were collected, which included demographics, comorbidities, presenting symptoms, pleural fluid analysis, data on chest tubes, intrapleural therapy, radiological data, the treatment offered, and the outcomes.

Subjects/procedure

All the patients had complicated parapneumonic pleural effusion or frank empyema. There were no exclusion criteria. CPPE was defined as pleural effusion with biochemical evidence of inflammation (pH < 7.2, glucose < 60 mg/dl, lactate > 1000 IU/l, and/or radiographic evidence of loculations). Empyema was defined as pus in the pleural space or positive gram stain and/or culture. Chest tubes were placed under ultrasound guidance in these patients, and the locations for thoracostomy were chosen using bedside ultrasound of the thorax. The choice of chest tube size was dependent on the attending physician's preference. The timing of tPA/DNase was based on the findings of loculations on the chest imaging and the amount of drainage in the first 24 hours. Daily tPA/DNase (5 mg DNase and 10 mg tPA, each in 50 ml of 0.9% sodium chloride) with separate syringes were administered intrapleural and concurrently. Concurrent tPA/DNase was injected in the chest tube, followed by a 50 ml flush. The tube was then clamped for 60 minutes followed by opening it to under water seal. The patient received doses in the morning at 8 a.m. and then at 5 p.m., administered by the physician. After two doses, a chest X-ray would be done to see the effectiveness (the area of pleural opacity on the chest X-ray was quantified by creating three-dimensional images). If the chest X-ray showed significant resolution, then a CT chest would be done for confirmation. The administration was stopped earlier if the patient had adverse effects (pain, bleeding).

Statistical analysis

Measures of central tendencies like mean and median were used as well to describe descriptive data.

## Results

A total of 38 patients (Table 1) were found to have been treated with concurrent tPA and DNase for complicated parapneumonic effusion or empyema. Fourteen were female (36.8%). The mean age was 61.4 years. There were 31 Caucasians, six African Americans, and one Asian American. Eight (21%) had a history of non-pulmonary malignancy, and 12 (32%) had a history of previous pulmonary disease (chronic obstructive pulmonary disease, asthma, and history of previous pneumonia). Many patients had a variety of medical conditions (Table 2). Of the effusions, 25 (65.8%) were located on the right side. A total of 29 (76.3%) effusions were loculated. The mean pleural pH was 6.84. There were 26 (68.4%) treated with chest tubes sized at 20 French or less. Pleural fluid cultures returned positive for 21 (55.3%), with the most common causative agents being methicillin-resistant *Staphylococcus aureus* (10.5%) and alpha-hemolytic streptococci (10.5%). Twenty (52.6%) patients received the full six doses of combined concurrent tPA/DNase. Of the 18 (47.4%) who did not, 11 did not require the full six doses for effusion resolution, and seven had to discontinue therapy due to tube blockage or pain. Only seven (18.4%) patients had complications related to tPA/DNase administration, most commonly pain (six patients complained of chest pain, while one had bleeding from pleural space, not requiring blood transfusion). Nineteen (50%) patients had complete radiological clearance of effusion, with 13 (34.2%) having partial clearance, and six (15.8%) having no change or worsening of their effusion. Eight (21.1%) patients needed further surgical management of their effusion.

**Table 1:** Demographics and clinical characteristics n: numbers; %: percentage; F: French; SD: standard deviation; tPA: tissue plasminogen activator; DNase: deoxyribonuclease.

Number of patients	38
Gender	
Female, n (%)	14 (36.8%)
Male, n (%)	24 (63.15%)
Race	
Caucasians, n (%)	31 (81.5%)
African Americans, n (%)	06 (15.7%)
Asians, n (%)	01 (2.6%)
History of malignancy, n (%)	08 (21%)
History of pulmonary diseases, n (%)	12 (31.5%)
Pleural effusion location	
Right pleural space, n (%)	25 (65.7%)
Left pleural space, n (%)	13 (34.2%)
Loculations, n (%)	29 (76.3%)
Mean pH (SD)	6.8
Chest tube size	
≤20 F, n (%)	26 (68.4%)
>20 F, n (%)	12 (31.5%)
Pleural fluid cultures positive, n (%)	21 (55.2%)
Number of tPA/DNase doses administered	
≤6 doses, n (%)	20 (52.6%)
>6 doses, n (%)	11 (28.9%)
Radiologic clearance	
Complete, n (%)	19 (50%)
Partial clearance, n (%)	13 (34.2%)
No change, n (%)	06 (15.7%)
Complications, n (%)	07 (18.4%)
Need for video-assisted thoracic surgery, n (%)	08 (21%)

**Table 2:** Medical comorbidities n: numbers; %: percentage.

Comorbidities	Number of patients
Depression, n (%)	04 (10.5%)
Anxiety, n (%)	06 (15.7)
Hypothyroidism, n (%)	03 (7.8%)
Diabetes mellitus, n (%)	10 (26.3%)
Gastroesophageal disease, n (%)	03 (7.8%)
Benign prostate hyperplasia, n (%)	02 (5.2%)
Hyperlipidemia, n (%)	07 (18.4)
Chronic kidney disease, n (%)	05 (13.1%)
Mitral valve prolapse, n (%)	01 (2.6%)
Hypertension, n (%)	01 (2.6%)
Osteoarthritis, n (%)	02 (5.2%)
Deep vein thrombosis, n (%)	01 (2.6%)

## Discussion

**Figure 1:**
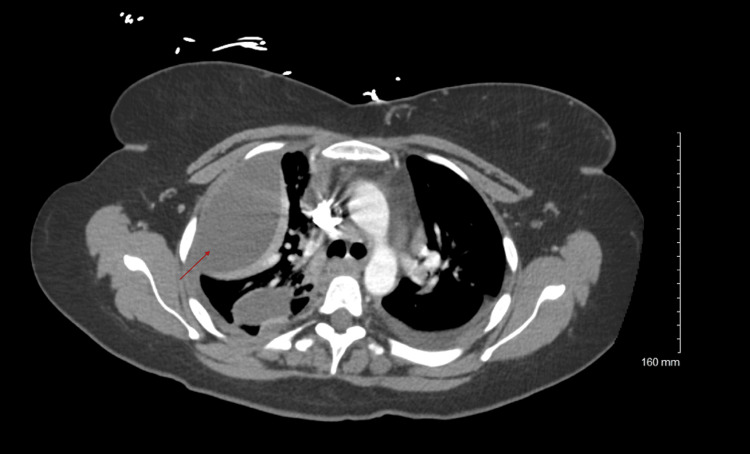
Loculation noted (red arrow) before tPA and DNase tPA: tissue plasminogen activator; DNase: deoxyribonuclease.

**Figure 2:**
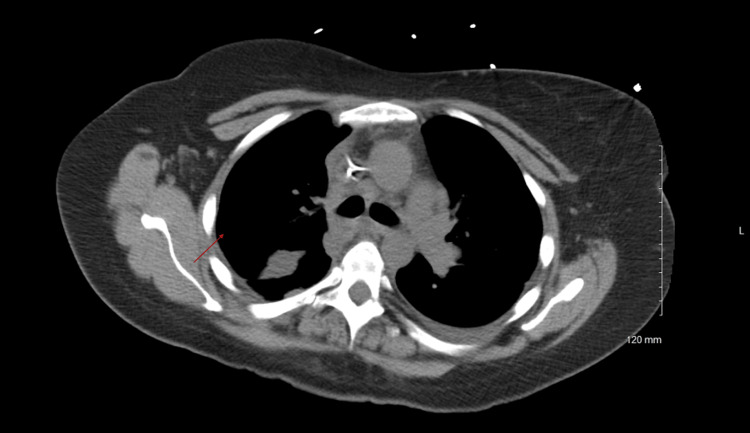
Resolution of loculation (red arrow) after the doses of tPA and DNase tPA: tissue plasminogen activator; DNase: deoxyribonuclease.

Our study shows that the concurrent treatment of pleural infection with tPA and DNase is safe and effective (Figures 1, 2). The optimal duration and dose of concurrent tPA/DNase use are unknown. The use of concurrent tPA/DNase has been well studied with encouraging results; however, there is little research elucidating the effects of dosing on therapeutic efficacy. The study results show that the current protocol of twice daily for three days dosing is not required by all patients for improvement of effusion. Likewise, the results from the study also suggest that some patients may require more than the typical six doses. The dosing regimen should be individualized depending on the clinical response of the patient.

The optimal combination (sequential vs. concurrent) and frequency of intrapleural therapy are not well established. The landmark study on sequential therapy, Multicenter Intrapleural Sepsis Trial (MIST2), was conducted by Rahman and colleagues [2], wherein 210 patients with pleural infections were randomly assigned to four study groups, one group being given sequential intrapleural tPA and DNase twice a day for three days. The dose of DNase was 5 mg and the dose of tPA was 10 mg and each administration was followed by clamping of the drain for an hour before releasing the clamp. Piccolo and colleagues [6,7] similarly did a multiple-center trial to look at the efficacy of sequential therapy in patients who failed initial conservative management with antibiotics and thoracostomy.

In a study done by Mehta et al. [8], 92% of patients who received sequential intrapleural therapy with tPA and DNase once daily were able to avoid surgical intervention. Popowicz and colleagues [9,10] experimented with different doses of DNase and tPA with positive outcomes.

Majid et al. [11] conducted a retrospective cohort study in patients who received concurrent tPA and DNase therapy for pleural infection. The overall treatment success rate was 90.4%, which was similar to the success rate of the study done by Piccolo and colleagues [6,7]. Our success rate was 79% with concurrent therapy; however, in the study done by Majid and colleagues, the treatment was started early (within 24 hours) as compared to our study when intrapleural therapy was not always started within 24 hours.

Kheir and colleagues [12] compared sequential vs. concurrent treatment with tPA and DNase (a total of 38 patients, 18 patients received tPA/DNase sequentially, and 20 patients received the dose concurrently); there was no statistically significant difference between the two treatment groups in median pleural fluid drainage or median volume of pleural effusion estimated on chest computed tomography scan.

Our study also confirmed the safety of concurrent intrapleural therapy, the rate of bleeding from pleural space was 5.4% in the study done by Majid et al. [11], whereas in our patient, only one patient had bleeding. The most common complaint was chest pain, 15% of our patients complained about it, which is comparable to the rate reported by Majid and colleagues [11]. Relative contraindication to fibrinolytic therapy is coagulopathy, the presence of bronchopleural fistula, and allergy to fibrinolytics [13]. Extra precaution should also be taken in patients with chronic kidney diseases, on concurrent anticoagulation therapy, and liver cirrhosis, as these conditions might increase the risk of bleeding [11].

Although the role of VATS is yet to be established, some of the recent studies [14,15] have had encouraging results, particularly if done early in the disease process (<4 weeks from symptoms onset) and in younger patients with low comorbidities. Federici and colleagues [15] similarly compared the early surgical vs. fibrinolytic approach. Although surgical management was associated with shorter chest tubes and hospital duration, the postoperative complication (postoperative arrhythmia and hemothorax) was higher. Additionally, fibrinolytic therapy was found to be more cost-effective compared to thoracoscopic decortication by Shipe and colleagues in their study [16].

Single center, retrospective nature, and small sample size were the limitations of our study. As such, further research is required to evaluate the optimal dose, frequency, and duration to improve drainage to further optimize patient outcomes.

## Conclusions

The results from our study suggest that concurrent dosing of tPA and DNase is safe and effective. It also suggests that some patients do not require the full six doses for improvement of effusion, or that some patients may require more than the typical six doses. Concurrent therapy is less burdensome (for the patient and medical staff), and therefore more attractive and practical option. Most of the research so far has pointed toward individualizing the dose and duration, which is consistent with our study results.
